# Novel Compound Heterozygous Splice-Site Variants in *TPM3* Revealed by RNA Sequencing in a Patient with an Unusual Form of Nemaline Myopathy: A Case Report

**DOI:** 10.3233/JND-230026

**Published:** 2023-09-08

**Authors:** Katarina Pelin, Lydia Sagath, Johanna Lehtonen, Kirsi Kiiski, Olli Tynninen, Anders Paetau, Mridul Johari, Marco Savarese, Carina Wallgren-Pettersson, Vilma-Lotta Lehtokari

**Affiliations:** a Molecular and Integrative Biosciences Research Programme, Faculty of Biological and Environmental Sciences, University of Helsinki, Helsinki, Finland; b Folkhälsan Research Center, Helsinki, Finland; c Department of Medical Genetics, Medicum, University of Helsinki, Helsinki, Finland; d Institute for Molecular Medicine Finland (FIMM), University of Helsinki, Helsinki, Finland; e Centre for Molecular Medicine Norway (NCMM), University of Oslo, Oslo, Norway; f Laboratory of Genetics, HUS Diagnostic Centre, Helsinki University Hospital and University of Helsinki, Helsinki, Finland; g Department of Pathology, University of Helsinki and Helsinki University Hospital, Helsinki, Finland; h Harry Perkins Institute of Medical Research, Centre for Medical Research, University of Western Australia, Nedlands, WA, Australia

**Keywords:** Nemaline myopathy, alpha-tropomyosin, TPM3, RNA sequencing, NMD-CGH array, linked-read sequencing

## Abstract

**Background:**

Pathogenic variants in the *TPM3* gene, encoding slow skeletal muscle *α*-tropomyosin account for less than 5% of nemaline myopathy cases. Dominantly inherited or *de novo* missense variants in *TPM3* are more common than recessive loss-of-function variants. The recessive variants reported to date seem to affect either the 5’ or the 3’ end of the skeletal muscle-specific *TPM3* transcript.

**Objectives:**

The aim of the study was to identify the disease-causing gene and variants in a Finnish patient with an unusual form of nemaline myopathy.

**Methods:**

The genetic analyses included Sanger sequencing, whole-exome sequencing, targeted array-CGH, and linked-read whole genome sequencing. RNA sequencing was done on total RNA extracted from cultured myoblasts and myotubes of the patient and controls. TPM3 protein expression was assessed by Western blot analysis. The diagnostic muscle biopsy was analyzed by routine histopathological methods.

**Results:**

The patient had poor head control and failure to thrive, but no hypomimia, and his upper limbs were clearly weaker than his lower limbs, features which in combination with the histopathology suggested *TPM3*-caused nemaline myopathy. Muscle histopathology showed increased fiber size variation and numerous nemaline bodies predominantly in small type 1 fibers. The patient was found to be compound heterozygous for two splice-site variants in intron 1a of *TPM3*: NM_152263.4:c.117+2_5delTAGG, deleting the donor splice site of intron 1a, and NM_152263.4:c.117 + 164 C>T, which activates an acceptor splice site preceding a non-coding exon in intron 1a. RNA sequencing revealed inclusion of intron 1a and the non-coding exon in the transcripts, resulting in early premature stop codons. Western blot using patient myoblasts revealed markedly reduced levels of the TPM3 protein.

**Conclusions:**

Novel biallelic splice-site variants were shown to markedly reduce TPM3 protein expression. The effects of the variants on splicing were readily revealed by RNA sequencing, demonstrating the power of the method.

## INTRODUCTION

Nemaline myopathies (NM) are a clinically and genetically heterogeneous group of congenital myopathies caused by autosomal dominant or recessive variants in one of at least 12 different genes. The clinical phenotype varies from severe, even lethal forms, to milder forms with onset in childhood. Typical clinical features include generalized muscle weakness, especially of the neck flexors, facial muscles, respiratory muscles, and proximal muscles of the limbs. Typical histological features include cytoplasmic nemaline rods derived from Z-disc proteins and predominance of small type 1 fibers [1, 2].

Pathogenic variants in *TPM3* (MIM: *191030) are a relatively rare cause of NM (NEM1, MIM: #609284), accounting for less than 5% of the cases, although *TPM3* was the first NM-causing gene discovered [3]. Besides NM, pathogenic variants in *TPM3* can cause congenital fiber type disproportion (CFTD, MIM: #255310) and cap myopathy (MIM: #609284) [4]. In *TPM3*, dominantly inherited or *de novo* missense variants are more common than recessive loss-of-function variants [5]. Recessive variants reported to date include nonsense, stop-loss, frameshift, splice-site, and missense variants [6–10]. We have previously described a large homozygous deletion removing the promoter and exons 1a and 2b of *TPM3*, resulting in a severe lethal form of NM [11].

There are four different tropomyosin-encoding genes in the human genome: *TPM1* (MIM: *191010), *TPM2* (MIM: *190990), *TPM3,* and *TPM4* (MIM *600317). Transcription from alternative promoters and alternative splicing of the tropomyosin genes generate more than 40 different isoforms expressed in various tissues and cell types. The tropomyosins may be divided into high-molecular-weight and low-molecular-weight tropomyosins. The high-molecular-weight tropomyosin is  284 amino acids long, and it is transcribed from the promoter preceding exon 1a in *TPM1*, *TPM2,* and *TPM3*. The low-molecular-weight tropomyosin is 247 amino acids long and transcribed from the promoter preceding exon 1b in all four genes [12]. The sarcomeric isoforms are of high molecular weight. All four tropomyosin genes are expressed in myoblasts. Expression of the skeletal muscle isoforms from *TPM1*, *TPM2,* and *TPM3* commences when the myoblasts fuse to form myotubes [13].

The *TPM3* gene encodes slow skeletal muscle *α*-tropomyosin, also known as *γ*-tropomyosin, which is expressed explicitly in slow muscle fibers, where it forms coiled-coil homodimers or heterodimers with β-tropomyosin (encoded by *TPM2*). In the sarcomere, the tropomyosin dimers bind head-to-tail along the actin filament and regulate muscle contraction together with the troponin complex [14]. Developmental expression studies using fetal and neonatal skeletal muscle samples showed that the expression of slow *α*-tropomyosin is very low during muscle development and reaches significant levels around birth [15]. Given that TPM3 is specific for type 1 slow muscle fibers, nemaline rods are almost exclusively seen in type 1 muscle fibers in patients with pathogenic variants in *TPM3* [2].

Here, we report compound heterozygous splice-site variants in intron 1a of *TPM3* associated with a severe form of NM with some unusual clinical features. Conventional RT-PCR and sequencing gave inconclusive results regarding the effect on splicing, but RNA sequencing (RNA-seq) revealed the mis-splicing events.

## CASE REPORT

### Clinical findings

This boy was born after an uncomplicated pregnancy at gestational week 39 + and his Apgar scores were 9, while the birth measurements were 3876 g/53 cm/35 cm. He was the third child of non-consanguineous, healthy parents and had two healthy brothers. Neonatally, he suffered an aspirational event, but otherwise, the immediate neonatal period was normal.

At the age of three weeks, he was admitted for hospital investigations because of feeding difficulties, vomiting, failure to thrive and muscle hypotonia. He had extremely poor head control but no hypomimia. On admission, he hardly had any voluntary movements, and when movements slowly started showing his upper limbs were clearly weaker than his lower limbs. A metabolic cause was sought for, and there was transient glyceroluria. At the age of three months, EMG showed normal findings. CK was 316 IU/l.

At the age of 11 months, the child was able to roll over, and his arms had gained some strength, but he still had very poor head control and generalized hypotonia. He showed severe weakness of the neck flexors, shoulder girdle muscles and abdominal muscles. In the supine position, he was unable to lift his head at all, but when provided neck support, he was able to turn his head to both sides, and in the prone position, with continued neck support, to extend his arms, raising his chest from the bed. The distal muscles of the upper limbs were of better strength than the proximal ones, and the infant was able to handle toys skillfully using both hands. His lower back muscles, gluteus maximus muscles and legs showed clearly better strength than the arms, extension being generally stronger than flexion. He was also able to raise his pelvis from the bed and to rotate, adduct, abduct and flex his hips. Joint movements were normal and there was no scoliosis. He had no hypomimia, but the palate was narrow and high-arched. Tendon reflexes were weak. His cognitive development appearednormal.

At the age of 13 months, the boy was able to eat and had no swallowing difficulties, but tired after a small meal. A gastrostomy was inserted at the age of 2 yrs 6 months because he had been able to feed without a tube for short periods only. There was still a significant discrepancy between his upper and lower limb strength, but his hand strength had improved and he was able to lift his arms above his head. His head control was still poor and he was to receive a headrest. His speech development was normal, but he spoke with a weak voice.

At the age of 1 yr 8 months, non-invasive night-time ventilation was initiated, but at the age of 2 yrs 9 months, the boy died unexpectedly at home of respiratory causes.

### Muscle histopathology

**Fig. 1:**
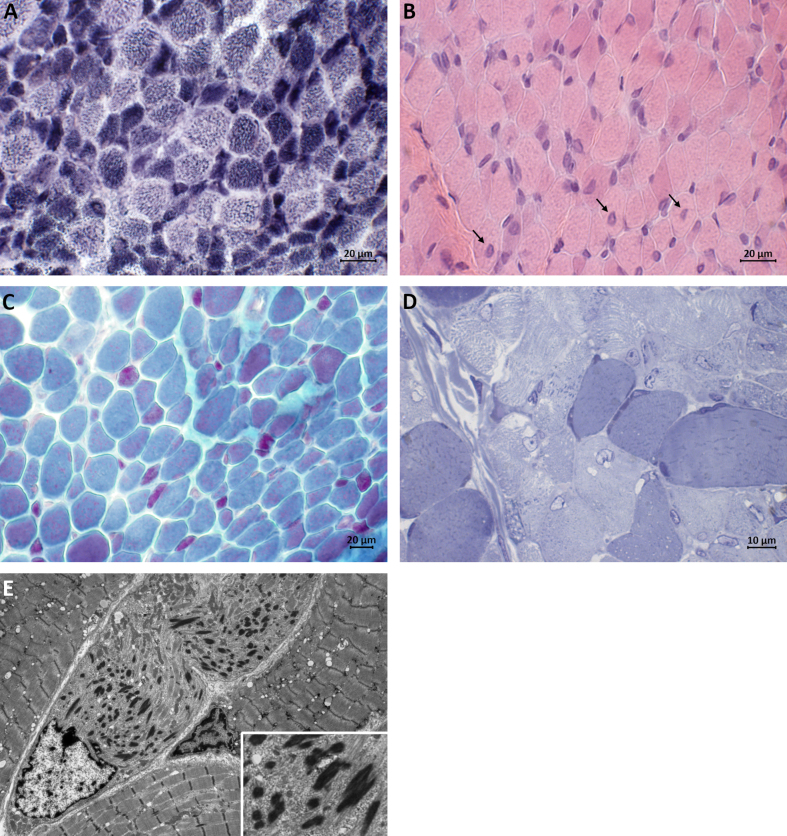
Muscle biopsy taken at the age of 7 months. (A) Darkly stained type 1 fibers are predominantly smaller in size than lightly stained type 2 fibers (NADH-TR staining). (B) H&E staining showing central nuclei in several fibers (arrows). (C) Red nemaline bodies are accumulated in small type 1 fibers and scattered rods are seen in larger fibers (Gomori trichrome staining). (D) Semithin section showing darkly staining rod-like structures (Toluidine blue staining). (E) Electron micrograph showing electron-dense ovoid-shaped nemaline rods.

A muscle biopsy was performed at the age of seven months. It showed prominent variation in fiber size, range 20–30μm. Numerous nemaline bodies were present at Gomori trichrome staining, predominantly in small type 1 fibers (fiber diameter approximately 10μm). Hypertrophic fibers (up to 30μm in diameter) were mainly of type 2, and some large undifferentiated fibers were present also. Haematoxylin-Eosin-stained sections showed central nuclei in several fibers while NADH-TR staining failed to reveal any cores. These findings were confirmed at electron microscopy, including myofibrillar disruption in small fibers, and led to a suspicion of *TPM3*-caused nemaline myopathy (Fig. 1).

### Genetic findings

The karyotype (G-banding) of the patient was normal. Initial-phase genetic testing for the *SMN1* deletion was negative.

DNA extracted from blood or cultured myoblasts of the patient was subjected to a number of genetic analyses including Sanger sequencing of *TPM3* as described in [16], whole-exome sequencing (WES) as described in [11], targeted array-CGH (NM- and NMD-CGH arrays) as described in [17, 18], and linked-read whole genome sequencing (WGS) as described in [19]. Sanger sequencing of parental DNA extracted from blood was used to establish the segregation of the variants in the family. RNA-seq was done on total RNA extracted from cultured myoblasts and myotubes of the patient and controls. Messenger RNA was purified using poly-T oligo-attached magnetic beads and used for library preparation (Novogene NGS RNA Library Prep Set, Novogene, Cambridge, UK). Sequencing was performed on a Novaseq 6000 PE150 (Illumina, CA, USA) generating nearly 18 G of raw data. Raw sequences were mapped with STAR 2.7.0d using the two-pass method [20] using the Gencode.v39 human genome reference assembly (based on GRCh38.p13). We compared the exon-exon junctions in *TPM3* in RNA-seq data of the patient with an internal control group (RNAseq data from muscle of over 200 myopathy and non-myopathy individuals). In addition, the sashimi plot representing the exon-exon junctions in *TPM3* was manually inspected using the Integrative Genomics Viewer (IGV).

**Fig. 2:**
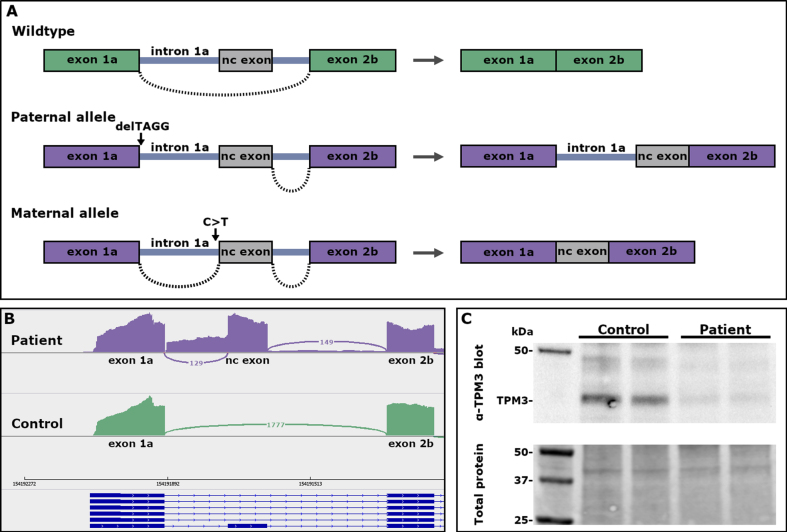
(A) A schematic representation of the effect of the *TPM3* variants on pre-mRNA splicing. (B) Sashimi plots showing the splicing pattern in the 5’end of *TPM3*. Inclusion of intron 1a and a non-coding exon in transcripts from myotubes of the patient, and normal splicing of exon 1a to exon 2b in control myotubes. (C) Western blot showing low expression of TPM3 in myoblasts from the patient.

The patient was compound heterozygous for two variants located in intron 1a of the *TPM3* gene. He had inherited a 4-bp deletion, NM_152263.4:c.117+2_117+5del (CADD: 29.2), deleting the donor splice site of intron 1a from his healthy father. There are four heterozygous carriers of the variant in gnomADv2.1.1 (https://gnomad.broadinstitute.org/, variant id: 1-154164372-GCCTA-G in GRCh37) and two heterozygous carriers in gnomADv3.1.2 (variant id: 1-154191896-GCCTA-G in GRCh38). The *in silico* splicing prediction tool SpliceAI [21] predicted loss of the donor splice site (delta score 0.97). RNA-seq revealed the inclusion of intron 1a in the transcript from this allele (Fig. 2A, B).

The patient had inherited a second *TPM3* variant, NM_152263.4:c.117 + 164 C>T (CADD: 9.62), from his healthy mother. This variant is also in intron 1a, located 5 bp upstream of a non-coding exon. SpliceAI predicted gain of an acceptor splice site (delta score 0.63). The non-coding exon contains two stop codons in frame with the coding sequence of *TPM3*. There is one carrier of this variant in gnomADv3.1.2 (variant id: 1-154191738-G-A in GRCh38). RNA-seq revealed inclusion of the non-coding exon in the transcript from this allele (Fig. 2A, B).

WES, WGS and the NMD-CGH array revealed additional rare variants in genes previously associated with congenital myopathies or genes with known high expression levels in skeletal muscle (Table 1). Heterozygous missense variants were identified in *RYR1* (MIM: *180901) and *YBX3* (also known as *CSDA*, MIM: *603437), and a hemizygous nonsense variant was identified in the X-chromosomal gene *SRPK3* (MIM: *301002). The premature stop codon in *SRPK3* would result in a protein lacking 100 amino acids from its C-terminus. Western blotting using the GTX111444 antibody (GeneTex, Irvine, CA, USA) on protein extracted from myoblasts and myotubes of the patient showed strong SRPK3 expression (results not shown).

**Table 1:** Rare variants of uncertain significance (VUS) in genes associated with congenital myopathies or in genes with high expression levels in skeletal muscle identified in the patient

Gene	Variant	Inheritance	REVEL	CADD
*RYR1* (MIM: *180901)	NM_000540.2:c.6093G>C,	Paternal	0.260	21.2
	NP_000531.2:p.(Leu2031Phe)			
*YBX3* (MIM: * 603437)	NM_003651.4:c.102C>A,	Maternal	0.033	22.2
	NP_003642.3:p.(Ser34Arg)			
	NM_003651.4:c.385C>T,	Paternal	0.393	24.5
	NP_003642.3:p.(Arg129Trp)			
*SRPK3* (MIM: *301002)	NM_014370.3 : c.1402G>T,	Maternal	NA	39
	NP_055185.2:p.(Gly468*)			
*DNPEP* (MIM: *611367)	20.1 kb duplication	Maternal	NA	NA
	Chr2:(19573512_219574571)_(219595787_218794896)dup (Hg38)			
*TNNT3* (MIM: *600692)	45.8 kb inversion	No data	NA	NA
	Chr11:(1894041_1894042)_(1939824_1939843)inv (Hg38)			

Abbreviations: NA, not applicable

The NMD-CGH array revealed a heterozygous 20.1 kb duplication spanning the 5'end of *DNPEP* (MIM: *611367), upstream of *OBSL1* (MIM: *610991). Linked-read WGS revealed a 45.8 kb inversion on chromosome 11 spanning *TNNT3* (MIM: *600692) and *LINC01150.* Several inversions in this region have been listed in the Database of Genomic Variants (http://dgv.tcag.ca/dgv/app/home).

### Western blot analysis

Whole cell extracts were prepared in RIPA buffer from cultured patient and control myoblasts, combined with 6x Laemmli buffer and heated at 98°C for 5 minutes. Equal quantities of total protein were applied to a Bio-Rad 4-20% gradient TGX mini gel (Bio-Rad Laboratories, CA, USA) and the gel run was performed at RT, 120 V, 90 minutes. Proteins were transferred onto PVDF membrane using the Bio-Rad TransBlot Turbo system (program Mixed SD, 7 min). Whole protein load was detected with Revert 700 Total Protein Stain (LI-COR Biosciences, Lincoln, NE, USA) on the Odyssey system (LI-COR Biosciences). The membrane was blocked in 5% milk in 0.1% TBST for 1 h. Mouse monoclonal antibodies against TPM3, (clone 3D5AH3AB4, ab113692, Abcam, Cambridge, UK) were incubated with the membrane at a 1 : 1000 dilution at +8°C for 18 h with gentle agitation. Polyclonal rabbit anti-mouse HRP-conjugated immunoglobulins (P0260, Agilent, Santa Clara, CA, USA) and ECL reaction (SuperSignal West Femto, Thermo Fisher Scientific) were used to detect the tropomyosin bands with the ChemiDoc MP digital imager (Bio-Rad).

The expression of a  30 kDa TPM3 molecule was consistently lower in all patient-derived samples run compared with the control myoblast extracts and when normalized for total protein load. The expression of TPM3 was roughly 45% of the expression in the control (Fig. 2C).

## DISCUSSION

Recessive variants in *TPM3* are rare and have hitherto mainly been seen in severe forms of NM or related congenital myopathies. The recessive loss-of-function variants in *TPM3* reported to date seem to affect either the 5’ or the 3’ end of the skeletal muscle-specific transcript encoding high-molecular-weight *α*-tropomyosin. The 5’ end variants include a homozygous nonsense variant in exon 1a in an infant with severe NM [6], a large homozygous deletion removing the promoter and exons 1a and 2b in a severe lethal form of NM [11], a homozygous point mutation in intron 1a, predicted to cause mis-splicing of exon 2a in a severe congenital myopathy [10], and the splice-site variants reported herein. The variants in all these cases abolish the expression of the muscle-specific transcript TPM3.12, but allow the expression of low-molecular-weight tropomyosin isoforms from the internal promoter of *TPM3*. The residual protein observed on Western blot (Fig. 2C) is probably due to leaky, normal splicing, most likely from the maternal allele. RNA-seq showed low levels of normal splicing in myotubes.

The clinical course of the patients described by Tan et al. [6] and Kiiski et al. [11] were similar to that of our patient, with severe presentation in the neonatal period, delayed and impaired motor development, and death before the age of 2 years.

The patient described by Yogev et al. [10] was diagnosed with a severe congenital myopathy with bilateral clubfeet, delayed motor development, hypotonia, and unusual features including tongue fasciculations and cerebral atrophy. The muscle biopsy showed fiber-type disproportion, and an increase in internal nuclei, but no nemaline rods, cores or caps. It is uncertain whether all the clinical features had been caused by his *TPM3* variant [10].

The recessive variants described hitherto at the 3’ end of *TPM3* are all located in exon 9b, i.e. the last exon of the skeletal muscle-specific transcript. In 2002, Wattanasirichaigoon and co-workers described a patient with NM of intermediate severity and compound heterozygosity for a stop-loss variant in exon 9b and a splice acceptor site variant causing skipping of exon 9b [7]. This stop-loss variant has also been found in homozygous form in a patient with CFTD [22], who became ventilator-dependent at the age of 2.5 yrs and who did not achieve walking. We have reported a recessive Turkish founder mutation in *TPM3*, i.e. a deletion of the last nucleotide before the stop-codon in exon 9b, resulting in a frameshift and read-through, and elongation of the protein by 73 amino acids. The homozygous patients had unusual forms of NM, with severe chest deformities or infantile scoliosis [16].

In comparison with NM patients who have the typical form [1], a few clinical features, similar to those in our patient, appear distinct among patients with recessively inherited *TPM3* myopathy. These are severe and prolonged feeding difficulties and/or failure to thrive [11, 22], severe respiratory problems and early need of a ventilator [10, 22], and very poor head control in comparison with the strength in other muscles [6, 9]. Interestingly, a clear discrepancy between the strength of the upper versus the lower limbs, as seen in our patient, and in [22], was also described in the original *TPM3* NM family with dominant inheritance of the variant p.Met9Arg in exon 1, published by Laing and coworkers [3]. However, in that family, weakness was more pronounced in the lower limbs, not in the upper ones, as in our patient.

Although most missense variants in *TPM3* cause dominant disease, a homozygous missense variant, NM_152263.4:c.535C>G, NP_689476.2:p.(Arg179Gly), has been reported to cause a mild phenotype of CFTD [9].

The rare variants identified in *RYR1*, *YBX3,* and *SRPK3* may have modified the phenotype of the patient described herein. During muscle maturation, dephosphorylated YBX3 accumulates in the nuclei of muscle fibers and represses myogenin and, thus, muscle differentiation [23]. Whether the missense variants identified in the patient have an impact on YBX3 function is the subject of future studies. The serine/arginine-specific protein kinase SRPK3 is also involved in the regulation of myogenesis by activating alternative splicing of the MEF2C*α*2 isoform that promotes myogenesis [24]. Our RNA-seq results did not reveal any differences in the alternative splicing of MEF2C exons *α*1 and *α*2 in myoblasts and myotubes from the patients and controls. The MEF2C*α*1 isoform was the predominant one in all samples, which is in accordance with the results published by Zhang and co-workers [24]. Therefore, it seems unlikely that the truncated SRPK3 affects myogenesis through MEF2C. However, the regulatory role of SRPK3 makes it a very likely modifier gene. The large structural variants affecting *DNPEP* and *TNNT3* are of unknown significance.

To the best of our knowledge, the patient reported here is the eighth index patient to be described with recessive variants in *TPM3* causing nemaline myopathy or a related congenital myopathy. Both variants in intron 1a were initially identified by targeted Sanger sequencing of *TPM3*, but the maternal variant was initially interpreted as benign. Only the paternal variant was identified by WES, whereas the deep intronic location of the maternal variant was not covered. Both variants were identified by WGS, but it was RNA-seq that finally solved the case. The effects of the variants on pre-mRNA splicing were readily revealed by RNA-seq, demonstrating the power of the method.

The study was approved by the Ethics Review Board of Helsinki University Hospital (number 195/13/03/00/11) and performed according to the Declaration of Helsinki.

The variants have been submitted to the LOVD database: TPM3.lovd.nl, RYR1.lovd.nl, CSDA.lovd.nl, SRPK3.lovd.nl, DNPEP.lovd.nl, TNNT3.lovd.nl.

## CONFLICT OF INTEREST

The authors declare that they have no known competing financial interests or personal relationships that could have appeared to influence the work reported in this paper.
